# The Influence of Spatial Location and Object Shape on Learning and Re-Learning Spatial Tasks in Dopamine Transporter-Deficient Rats

**DOI:** 10.3390/biology15161363

**Published:** 2026-08-11

**Authors:** Natalia Kurzina, Anastasia Belskaya, Camilla Gabaydulina, Polina Sylko, Raul R. Gainetdinov, Anna Volnova

**Affiliations:** 1 Institute of Translational Biomedicine, Saint Petersburg State University, Saint Petersburg 199034, Russia; n.kurzina@spbu.ru (N.K.); a.belskaya@spbu.ru (A.B.); st101564@student.spbu.ru (P.S.); r.gainetdinov@spbu.ru (R.R.G.); 2 Biological Faculty, Saint Petersburg State University, Saint Petersburg 199034, Russia; st118207@student.spbu.ru

**Keywords:** dopamine, dopamine transporter (DAT), DAT-KO rats, attention deficit hyperactivity disorder (ADHD)

## Abstract

**Background:** The role of dopamine in brain functions has been well established, and dopamine imbalances can cause problems in working memory and spatial learning. The dopamine transporter (DAT) plays a significant role in dopamine regulation. Rats with a knockout of the dopamine transporter gene (DAT-KO), the model of attention deficit hyperactivity disorder (ADHD), are characterized by hyperdopaminergia and spatial memory impairments. **Methods:** Rats were trained to manipulate objects and recognize their spatial location and shape using RedBox apparatus. They needed to move a distant object to receive a food reward. After changing the shape and spatial location of objects, the ability to re-learning was evaluated. **Results:** We demonstrated that including objects to manipulate in the task conditions enables faster and stable learning of DAT-KO rats. The shape of the object did not influence the level of task performance, but changing the spatial location of the object led to the impairment of task performance in hyperactive rats. **Conclusions:** The data obtained highlight the role of hyperactivity and hyperdopaminergia in DAT-KO rats in spatial learning. We suggest that our findings may help in the further development of new training tasks for individuals with ADHD and spatial memory deficits.

## Simple Summary

People with attention deficit hyperactivity disorder (ADHD) often experience difficulties with memory and learning, especially when they need to remember the spatial arrangement of objects. The aim of this study was to better understand these problems using rats with knockout of the dopamine transporter gene that exhibit hyperactive behaviors similar to those in patients. We tested whether manipulating and moving objects could improve learning in hyperactive rats when the shape or location of objects changed. The results showed that the rats performed better in tasks involving the search for food rewards when they were able to manipulate the objects. The results suggest that incorporating object manipulation may help improve spatial learning in people with attention deficit hyperactivity disorder. The translation potential of data obtained could contribute to the development of more effective educational and rehabilitation strategies that make learning easier and more engaging.

## 1. Introduction

Adaptation to changes in the environment is essential for survival, especially in cases of neurological and neuropsychiatric diseases. Spatial memory and spatial orientation are necessary for finding food and safe places. This type of memory provides efficient navigation towards desired destinations and increases levels of adaptation. Disturbances in spatial memory are found in different diseases caused by abnormal dopamine (DA) function. Spatial memory and spatial orientation impairments have been found to be associated with a number of neurodegenerative and neurological diseases, including Alzheimer’s disease, Parkinson’s disease, and attention deficit hyperactivity disorder (ADHD). Patients with Parkinson’s disease exhibit difficulties in navigation and cognitive flexibility due to the degeneration of dopaminergic neurons in the substantia nigra [1,2,3], and spatial memory deficits are observed even in patients with mild clinical symptoms [4]. Impaired spatial memory is also observed in Alzheimer’s disease [5]. In patients with ADHD, impairment of attention, working memory, and other cognitive deficits have been described [6]. Long-term studies of adolescents with ADHD symptoms have shown that these patients demonstrate poorer performance than their peers in spatial tasks [7]. Similar impairments in spatial contextual memory have also been identified in adults with ADHD symptoms [8], and now it is generally accepted that ADHD is prevalent not only among children but also among adults [9,10].

The monoamine neurotransmitter DA regulates many important behavioral functions, such as movement, motivation, emotional responses, learning, and memory in animals and humans [11]. Disruptions in dopaminergic system functions can lead to impairments in motor and cognitive functions [12]. Numerous studies indicate that dysfunction in DA regulation leads to complex neurobehavioral impairments, including learning and memory deficits [13,14,15]. The abnormal functioning of the dopaminergic system also leads to enhanced motor hyperactivity and inattention. All these features contribute to decreasing adaptive behavior [15].

Traditionally, spatial learning has been associated with activity in the hippocampal-entorhinal system [16]. Unconditioned stimuli associated with a reward activate DA neurons that project to the nucleus accumbens (NAc) [17]. It has been demonstrated that the combination of dopaminergic and hippocampal glutamatergic inputs to this brain region is responsible for the formation of spatial memories associated with the attainment of goals [18,19].

The DA transporter (DAT) plays a crucial role in regulating dopaminergic transmission under normal and pathological conditions [20]. It removes DA from the synaptic cleft back into the presynaptic neuron for reuse, thereby terminating its action on the relevant receptors and limiting its effects [21,22]. DAT is encoded by the *SLC6A* gene, and numerous studies have examined its role in regulating the release and reuptake of DA [22,23,24,25]. Several studies have shown that variations in the *SLC6A3* gene are associated with structural differences in the right lateral prefrontal and cingulate cortical regions of patients with ADHD [26].

There are different approaches to memory and learning investigations in DA system dysfunction using experimental animal models. To model DAT dysfunction, mice lacking the *SLC6A* gene, which results in DAT deficiency, were created [27,28].

These animals exhibit behavioral abnormalities [29], including hyperactivity, impulsivity and perseveration, accompanied by cognitive impairment [30,31]. Therefore, these mice provide a model of the major endophenotypes of human diseases such as ADHD [32,33].

Compared to mice, rats are a more preferable model for studying cognitive impairments because they are capable of learning more complex behavioral tasks than mice. Around 10 years ago, a rat line with a knockout *SLC6A3* gene (DAT-KO rats) was created [34]. These animals exhibited the core symptoms of ADHD, including hyperactivity, reduced attention, impulsivity, and stereotypical behavior [27]. In previous experiments, we investigated the ability of DAT-KO rats, i.e., a model of ADHD, to solve orientation-related tasks in different types of mazes. We found that DAT-KO rats could perform such tasks; however, they were less efficient than WT animals and used different maze navigation strategies from control rats [35]. When performing spatial navigation tasks without additional cues, such as in the Hebb–Williams maze, DAT-KO rats were significantly less successful than wild-type rats and performed the task less efficiently [36,37,38].

DAT-KO rats exhibit not only pronounced motor hyperactivity but also perseverative behavioral patterns, such as trajectory repetitions, returns to the starting box, and stereotypical movements [39]. These patterns may prevent the animals from focusing on task performance [40]. However, the inclusion of objects for manipulation into the task paradigm helped to interrupt animals’ hyperlocomotion and slow their running rate, leading to more effective task performance. We found that the most successful learning was observed in manipulative tests [40,41]. In this study, we used a spatial orientation task with various spatial arrangements of objects to manipulate performance for the obtainment of rewards. We compared the importance of an object’s shape versus its spatial location for effective learning and re-learning in DAT-KO rats. Our hypothesis was that incorporating information about objects’ shape and using manipulative skills could help rats with spatial memory impairments to perform tasks successfully.

## 2. Materials and Methods

### 2.1. Animals

In our experiments, we used littermate DAT-KO rats (n = 11) and control wild-type (WT) rats (n = 10), which were 3–6 months old and weighed 250–300 g at the beginning of the experiments. The rats in the control and experimental groups were littermates. The knockout animals carried the mutation in a homozygous state (DAT−/−). The control group rats (WT) from the same litters did not carry the mutation in this gene (DAT+/+). Prior to the experiments, the rats were housed in IVC cages (RAIR IsoSystem World Cage 500, Lab Products, Inc., Aberdeen, MD, USA ) and had unlimited access to food and water. The animals were kept at a temperature of 22 ± 1 °C with a humidity level of 50–70%. The colony room was illuminated with artificial light from 9 a.m. to 9 p.m.

For genotyping, we used ear tissue samples from 15-day-old rats. Genomic DNA extraction was performed according to the alkaline method protocol and included two stages: polymerase chain reaction (PCR) and enzymatic digestion using a specific restriction enzyme [34]. The *Slc6A3* gene, which encodes a dopamine transporter, contains a specific nucleotide sequence that serves as a substrate for restriction. The mutation introduced in DAT-KO rats leads to the loss of this restriction site. As a result, the WT rats do not carry any mutant alleles. DAT-het rats retain only one intact allele, while knockout rats carry two mutant alleles.

Before the experiment began, the animals were food-restricted for a week, resulting in a weight loss of around 10%. Their weight was monitored daily throughout the experiment by weighing, and the amount of food they received was adjusted accordingly. All experiments were conducted during the light phase of the light/dark cycle, between 1 p.m. and 5 p.m. The experiments were conducted in compliance with “The Regulations on Research Using Experimental Animals” (Order of Ministry of Health of USSR #742 of 13.11.1984), FELASA, and RusLASA requirements regarding the care and treatment of laboratory animals. Approval was obtained from the Ethics Committee of Saint Petersburg State University, No. 131-03-6 of 25 April 2025.

### 2.2. Apparatus and Objects Used

**Figure 1:**
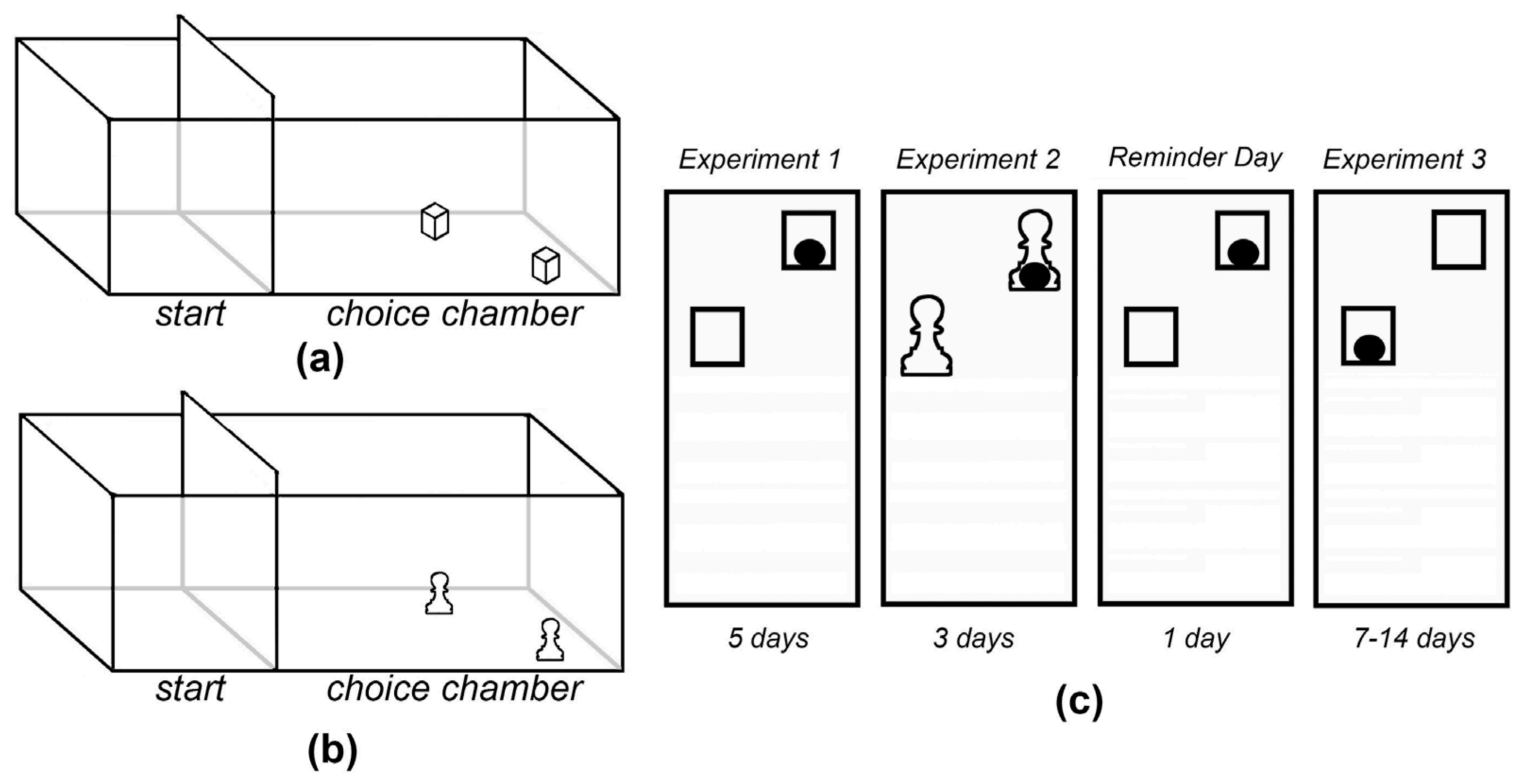
The construction of the RedBox apparatus with the start and choice chamber and various objects: (**a**) with cubes and (**b**) with pawns. (**c**) Stages of the experiment with variants of the mutual arrangement of the reinforced (food reinforcement is indicated by a black dot) and non-reinforced objects. Experiment 1 lasted 5 days, Experiment 2 lasted 3 days, and Experiment 3 lasted from 7 to 14 days. Success in re-learning was defined as achieving 80% correct runs in trials.

The RedBox testing apparatus was designed following on R.P. Kesner’s experiments [42,43]. The apparatus consisted of a rectangular arena 125 cm in length and 40 cm in width with a matte black floor and 40 cm high non-transparent red Plexiglas walls (Figure 1a). The arena was divided into two separate compartments by a removable, 40 × 25 cm red Plexiglas guillotine door. Rats began each trial in the start chamber (40 × 40 cm), and the objects were presented in the larger compartment (85 × 40 cm). A 6 × 6 matrix of ‘food wells’ (diameter: 2 cm; depth: 1 cm) was drilled into the floor at the end of the choice chamber. The rows and columns of wells were separated by 2.5 cm.

The pre-training period included one week of handling for 30 min a day and familiarization of the animals with the test apparatus.

Before the experiment began, there was a period of adaptation of animals to the apparatus. Each animal was placed in the starting area, and then it moved into the experimental area, where for 5 min it could explore the arena with objects and reinforcement that were freely available. The animals were also trained to leave the experimental area on their own after receiving rewards before then returning to the start box. During the experiment, the animal was placed in the start chamber; then, after raising the guillotine door, it could run to the choice chamber to search for food. After receiving reinforcement, the animal returned to the starting area, and the door was closed.

In terms of objects, two white wooden cubes of the same color (white), weight (40 g), and dimensions (5 × 4 × 4 cm) were placed to cover two near or distant wells in the choice chamber (Figure 1a). Moreover, two pawns of the same color, weight, and dimensions were placed at the same positions (Figure 1b).

### 2.3. Task Procedure

Rats were trained to find a food reward hidden under the object within a choice chamber. Rats were placed in a start chamber and ran into a choice chamber to perform the task; every day, each animal made 10 runs.

To obtain reinforcement, rats had to manipulate objects by moving them out of the wells. Small pieces of Popcorn Breakfast Loops (Khrutka, Nestle Russia LLC, Moscow, Russia) weighing 0.2 g were used as food reinforcement. A food reward was placed under only one of the two objects. The animals were trained to move the rewarded object to obtain food and not to move the non-reinforced object (the one with no food reward). An animal was considered to have learned the task rules if it performed at least 80% correct attempts, resulting in reinforcement, for three days in a row.

In Experiment 1, rats were presented two cubes, and the reinforcement was placed under the distant one (Figure 1c). During this experiment, the rats were trained to move only the distant cube to obtain food reinforcement and not to move the nearest cube, before then returning to the start chamber. The training lasted 5 days.

In the next stage (Experiment 2), the shape of objects was changed. Instead of cubes, we used pawns that were placed at the same positions, and the reinforcement was also located under the distant object. This training lasted 3 days (Figure 1c). After Experiment 2 with the new objects, rats were reminded of the conditions of Experiment 1 for one day (Figure 1c, Reminder Day).

Experiment 3 (Figure 1c) aimed to investigate the ability of experimental animals to re-learn. The food reinforcement at this stage was under the nearest cube, and the interposition of the cubes remained the same. The animal had to learn to move only the nearest cube to obtain reinforcement (and ignore the distant one). Since the success of re-learning was assessed when a correct rate of 80% was achieved in trials, the duration of this stage varied for different animals and ranged from 7 to 14 days.

### 2.4. Recorded Behavioral Parameters

The behavior of the rats was recorded and analyzed using a video tracking system and EthoVision XT software, ver.11.5.1026 (Noldus Information Technology, Leesburg, VA, USA). The configuration of the tracks followed by the rats during exploration of the choice chamber and search for reinforcement was analyzed. The following behavioral parameters were measured: the time spent in the choice chamber of the apparatus and the distance traveled by rats during the task. For analysis of the dynamics of learning, we also analyzed the percentage of animals that reached the learning criterion (at least 80% correct trials on 3 consecutive days) during learning and re-learning.

All animal responses were divided into four types: “correct runs” and the three types of “error runs”. The “correct runs” mean that the animal moved only the rewarded object. We specifically analyzed the different types of “error runs” that animals made when exploring the choice chamber and searching for reinforcement. All errors were divided into several types: (1) the rat moved both objects; (2) the rat moved only the non-reinforced object; and (3) the rat did not move any objects during the choice chamber exploration. The percentage of each response type for each rat was calculated from the total number of responses.

### 2.5. Statistical Analysis

The data were presented as the means ± SEM; *p* < 0.05 was considered statistically significant for all tests. A preliminary estimation of the data distribution normality (Gaussian distribution) was performed using the Kolmogorov–Smirnov test. We used the Mann–Whitney test, the ordinary one-way ANOVA test with Holm–Sidak’s multiple comparisons test, or the Kruskal–Wallis test with Dunn’s multiple comparisons post-hoc test. We used a log-rank Mantel–Cox test to compare the percentage of animals that reached the learning criterion on a given day of the experiment. All results were analyzed using GraphPad Prism 8 (GraphPad Soft-ware, Inc., San Diego, CA, USA).

## 3. Results

### 3.1. Experiments 1 and 2: Training Rats to Recognize the Objects’ Shape and Position

**Figure 2:**
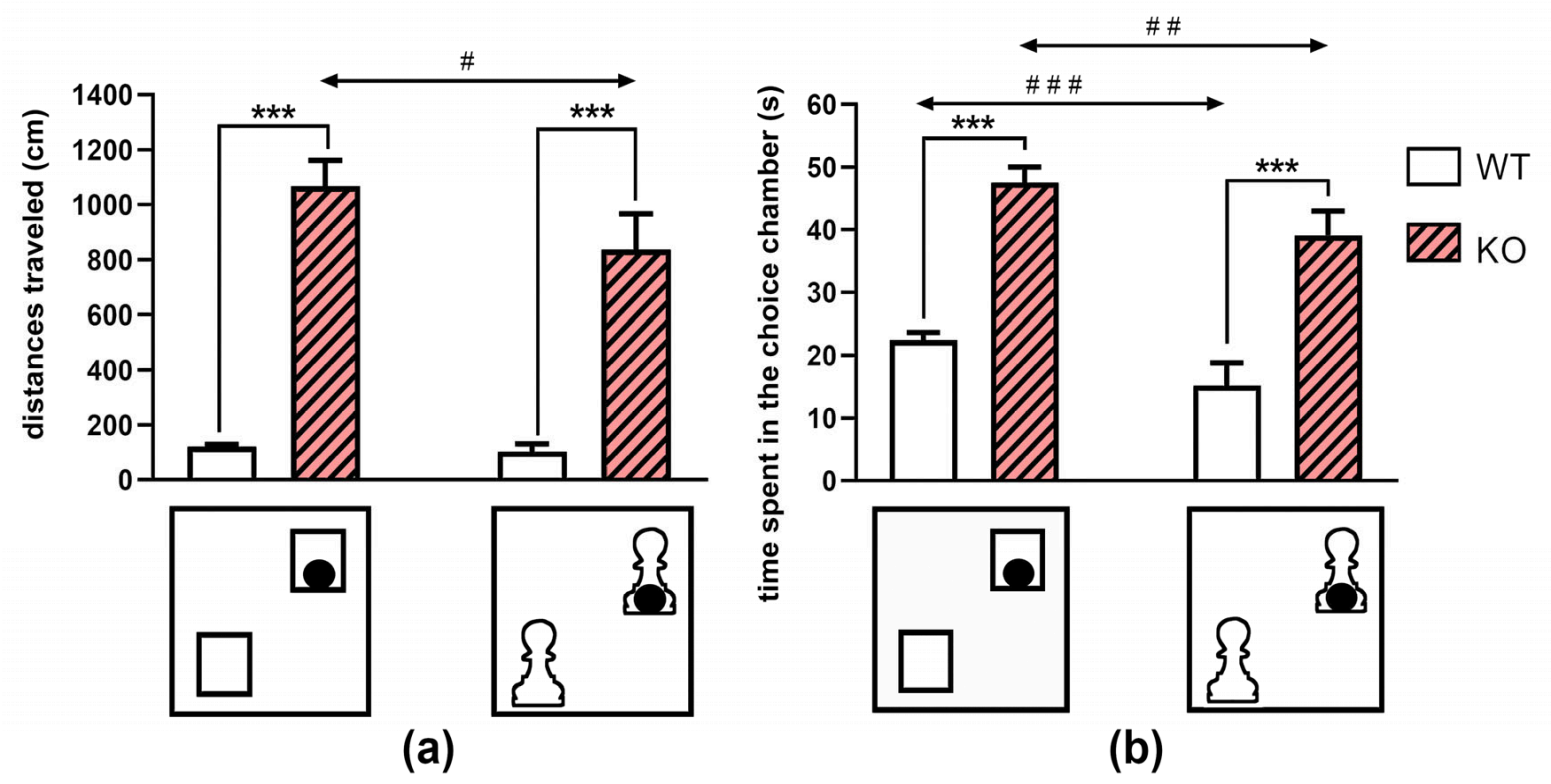
Distances traveled (**a**) and time spent in the choice chamber (**b**) by WT (n = 10) and DAT-KO (n = 11) rats during learning when cubes or pawns were presented. Results are presented as the mean ± SEM. # *p* < 0.05; ## *p* < 0.01; ### *p* < 0.001; Kruskal–Wallis test with Dunn’s multiple comparisons; *** *p* < 0.001, Mann–Whitney test. The reinforced objects are indicated by black dots.

In a series of experiments, rats were trained to discriminate the position of an object in a choice chamber by choosing a more distant cube (Experiment 1) or a pawn (Experiment 2, see Materials and Methods, Section 2.2.). First, we compared the distances traveled by WT and DAT-KO rats during learning. As usual, DAT-KO rats traveled significantly longer distances than WT rats (Kruskal–Wallis test with Dunn’s multiple comparisons, *p* < 0.001) and spent significantly more time in this experimental paradigm (the same test, *p* < 0.001) independently of the object shape (Figure 2a,b).

It should be noted that in WT rats, no differences were found in distances traveled in Experiment 1 and Experiment 2 (Kruskal–Wallis test with Dunn’s multiple comparisons, *p* = 0.057), whereas the time of experiment was significantly shorter during pawns’ presentation than in cubes (Kruskal–Wallis test with Dunn’s multiple comparisons, *p* < 0.0001). For DAT-KO rats, both of these parameters reduced after changing the object shape from cubes to pawns (Figure 2a,b). There were significant differences when comparing the distance traveled (Kruskal–Wallis test with Dunn’s multiple comparisons, *p* = 0.013) and the time spent completing the task (Kruskal–Wallis test with Dunn’s multiple comparisons, *p* = 0.003).

Thus, it is evident that DAT-KO rats explore the choice chamber in a different way than wild-type animals. They traveled a longer distance (Mann–Whitney test, *p* < 0.001) and, consequently, spent more time (Mann–Whitney test, *p* < 0.001) solving the task than control animals.

**Figure 3:**
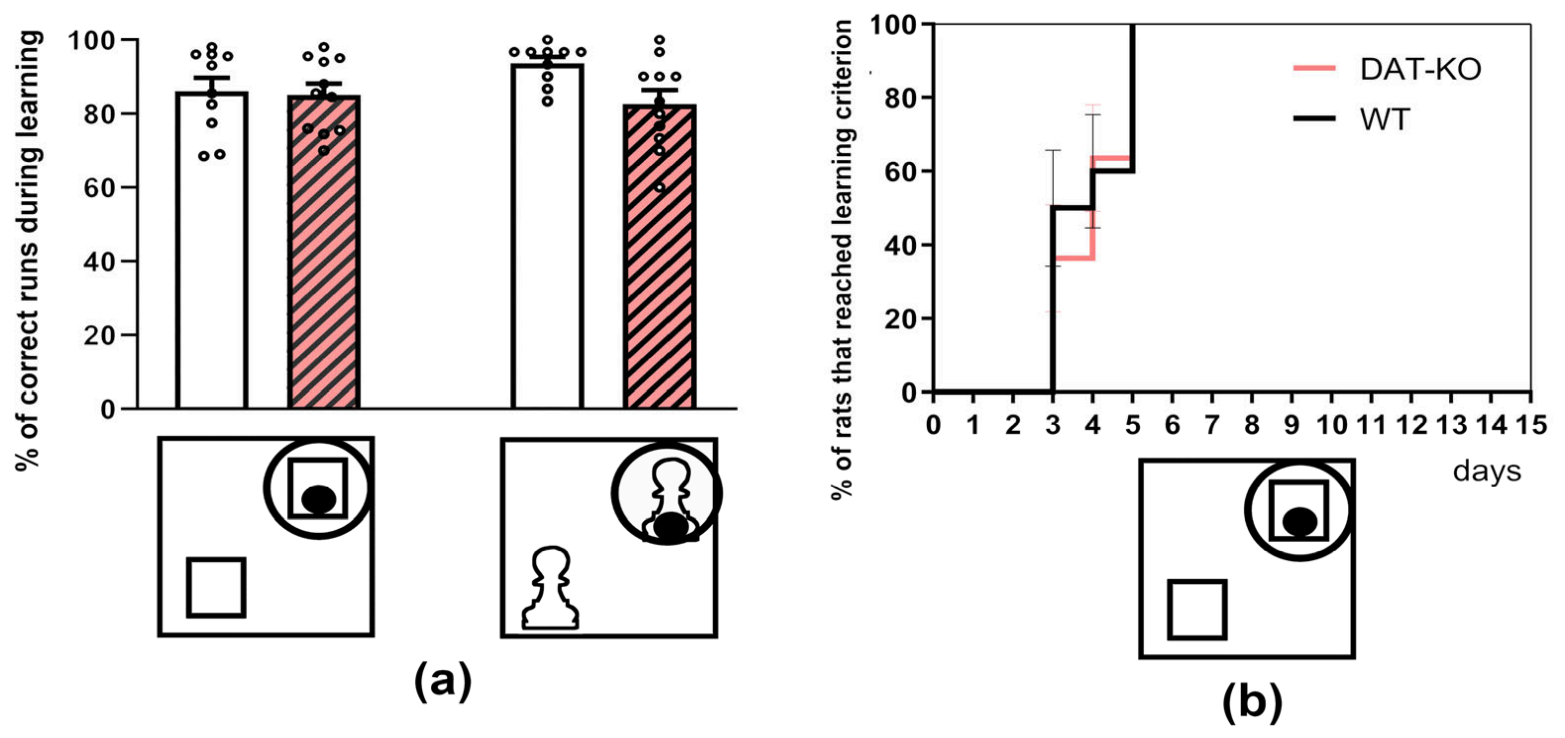
Comparison of the (**a**) percentage of correct runs during learning in WT (n = 10) and DAT-KO (n = 11) rats when cubes or pawns were used; WT—white bar, DAT-KO—red bar; results are presented as the mean ± SEM; Kruskal–Wallis test with Dunn’s multiple comparisons, no significant differences were found. (**b**) Number of animals (in percentage) who reached the learning criterion on a particular day of the experiment; the abscissa shows the days of the experiments; the ordinate shows the percentage of rats. In comparison of “survival” curves using the log-rank (Mantel–Cox) test, no significant differences were found. The designation of reinforced and non-reinforced objects is as shown in Figure 2. The object selection option is marked by a circle.

First, analysis of the results of Experiment 1, in which the food reward was placed under the distant cube, showed that DAT-KO rats performed tasks at the same level as WT rats. A comparison of the percentage of correct reactions in DAT-KO rats (86.15%) and WT rats (85.14%) is presented in Figure 3. No significant differences were found for this parameter (ordinary one-way ANOVA with Holm–Sidak’s multiple comparisons, *p* = 0.822). The dynamics of learning in WT and DAT-KO rats in Experiment 1 also showed no differences in this parameter (Log-rank Mantel–Cox test, *p* = 0.974) (Figure 3b). On the fifth day of training, all rats in both the knockout and control groups reached the learning criterion.

After changing the object from a cube to a pawn (Experiment 2), rats in both groups quickly learned the new conditions of the task. The percentage of correct responses in DAT-KO rats (93.67%) and WT rats (82.73%) remained at the same level and did not differ significantly in comparison with Experiment 1’s results (ordinary one-way ANOVA with Holm–Sidak’s multiple comparisons, *p* = 0.068) (Figure 3a). A diagram of the dynamics of rat learning by day was not constructed (Figure 3b) since all animals, both DAT-KO and WT, reached the learning criterion on the third day of the experiment (Log-rank Mantel–Cox test, *p* = 0.94).

Thus, it was demonstrated that the training of DAT-KO animals in the task of searching for food reinforcement under a distant object was as effective as in WT. The efficiency of finding food rewards in DAT-KO and WT rats in Experiments 1 and 2 also did not differ significantly (Figure 3). Changing the object’s shape from a cube to a pawn did not affect task performance.

**Figure 4:**
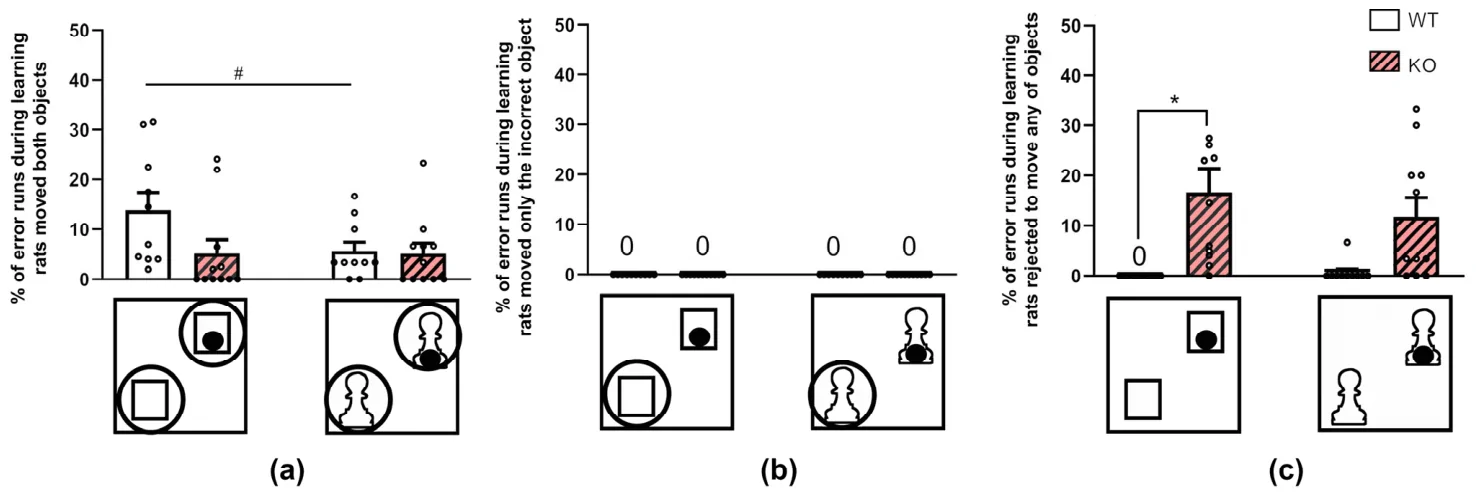
Comparison of the percentage of error runs during learning in WT (n = 10) and DAT-KO (n = 11) rats when cubes or pawns were used; (**a**) animals moved both objects; (**b**) animals moved only the incorrect object and (**c**) animals refused to move any of the objects. Results are presented as the mean ± SEM. # *p* < 0.05; * *p* < 0.05; Kruskal–Wallis test with Dunn’s multiple comparisons. The designation of reinforced and non-reinforced objects is as shown in Figure 2. The object selection option is marked by a circle.

We observed three different types of error runs. The animals could move both objects (first-type error), move only the incorrect object (second-type error), and even refuse to move any of them (third-type error). The number of first-type errors (Figure 4a) did not significantly differ in DAT-KO and WT groups of rats (Kruskal–Wallis test with Dunn’s multiple comparisons, *p* > 0.999 and *p* = 0.295 respectively). Changing the object from a cube to a pawn did not alter the percentage error rate for this type of errors in knockout rats (5.15%), but it reduced the error rate in WT rats (from 13.9% to 5.8%, Kruskal–Wallis test with Dunn’s multiple comparisons *p* = 0.02). Analysis of the second type of error showed that, during all periods of learning, the rats did not move only incorrect objects, irrespective of their shape (Figure 4b). However, the rate of third-type error, specifically, the percentage of errors in which the rats failed to move any objects, was significantly higher in DAT-KO rats (16.69% for cubes, Kruskal–Wallis test with Dunn’s multiple comparisons *p* = 0.03; and 11.82% for pawns) in comparison WT rats, in which such errors were practically absent (Figure 4c).

### 3.2. Experiment 3: Re-Training Rats to Recognize Objects’ Position (Re-Learning)

At this stage of the study (Experiment 3), the rats’ ability to re-learn the spatial location of an object associated with food reinforcement was evaluated. The results showed that DAT-KO rats were capable of re-learning and receiving reinforcement by moving the nearest cube.

**Figure 5:**
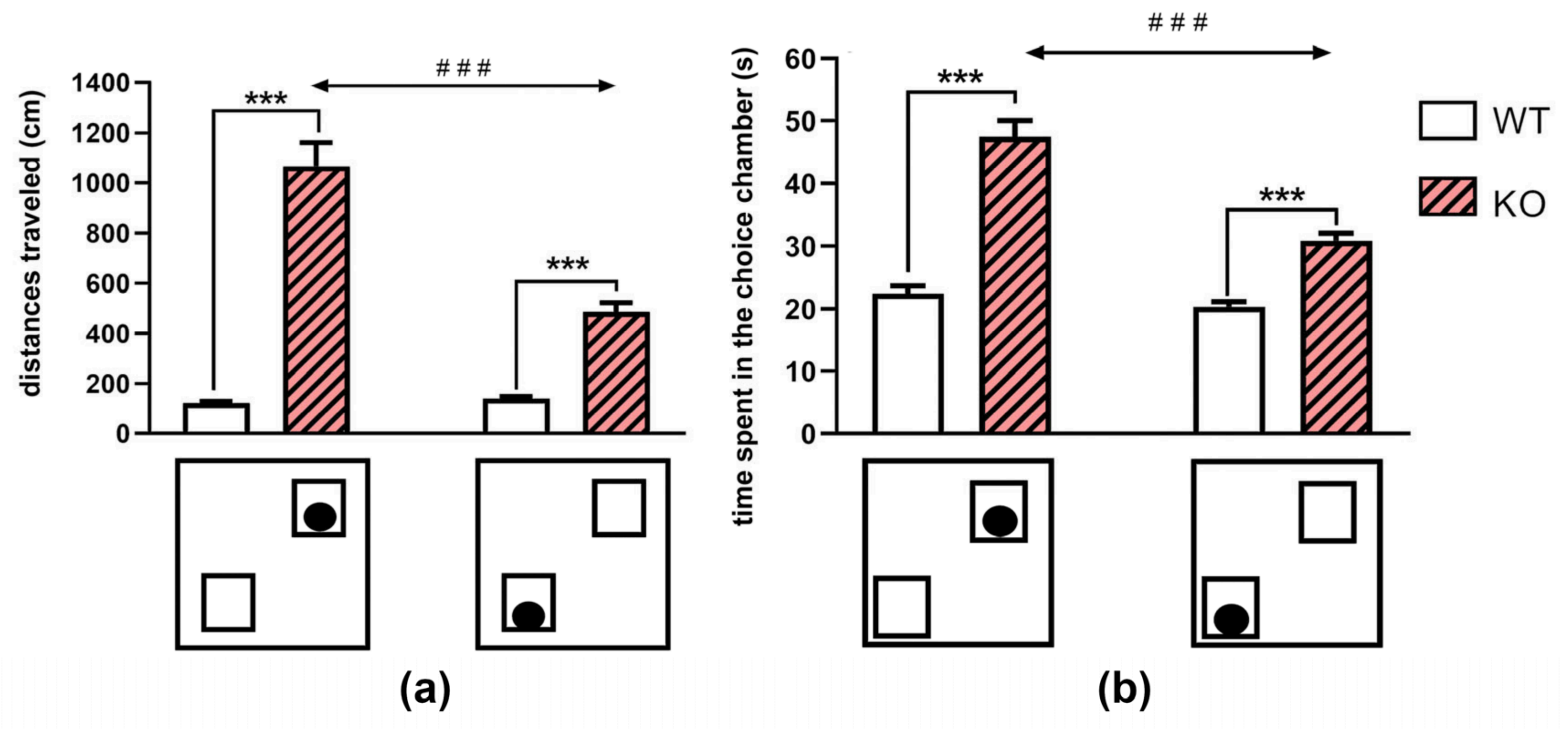
Distances traveled (**a**) and time spent in the choice chamber (**b**) in WT (n = 10) and DAT-KO (n = 11) rats during re-learning when the spatial position of the objects was changed. Results are presented as the mean ± SEM. ### *p* < 0.001; *** *p* < 0.001; Kruskal–Wallis test with Dunn’s multiple comparisons. The designation of reinforced and non-reinforced objects is as shown in Figure 2.

As in the previous experiments, the DAT-KO rats traveled a longer distance and spent more time exploring the choice chamber in comparison to WT rats (Mann–Whitney test, *p* < 0.001). However, when the behavioral parameters during re-learning and learning were compared in DAT knockout animals, a significant decrease in distance travelled (Kruskal–Wallis test with Dunn’s multiple comparisons, *p* < 0.001) and time spent in the choice chamber (Kruskal–Wallis test with Dunn’s multiple comparisons, *p* < 0.001) was found in the experiment (Figure 5a,b). For WT rats, there were no significant changes in these parameters.

**Figure 6:**
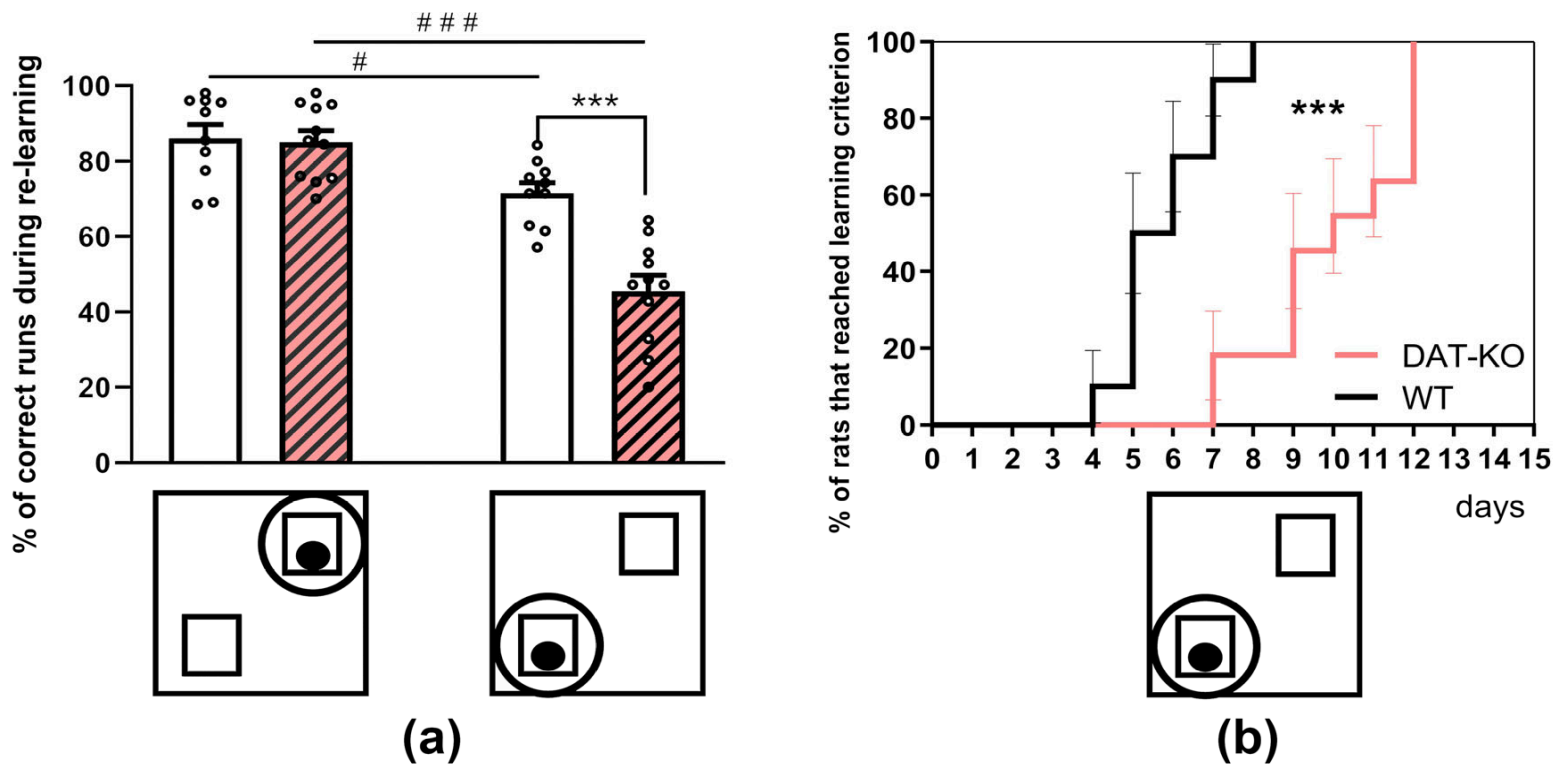
Comparison of the (**a**) percentage of correct runs during re-learning in WT (n = 10) and DAT-KO (n = 11) rats when the spatial position of the objects was changed; WT—white bar, DAT-KO—red bar; results are presented as the mean ± SEM. # *p* < 0.05; ### *p* < 0.001; *** *p* < 0.001; ordinary one-way ANOVA with Holm–Sidak’s multiple comparisons. (**b**) Number of animals (in percentage) who reached the re-learning criterion on a particular day of the experiment; the abscissa shows days of the experiments; the ordinate shows the percentage of rats; comparison of “survival” curves using the log-rank (Mantel–Cox) test, *** *p* < 0.001. The designation of reinforced and non-reinforced objects is as shown in Figure 2. The object selection option is marked by a circle.

Evaluation of the re-learning efficiency revealed that the percentage of correct reactions during the task decreased in both WT (from 86.15% to 71.57%, ordinary one-way ANOVA with Holm–Sidak’s multiple comparisons, *p* = 0.012) and DAT-KO rats (from 85.14% to 45.45%, ordinary one-way ANOVA with Holm–Sidak’s multiple comparisons, *p* < 0.0001) (see Figure 6a). At the re-learning stage, the DAT rats were less successful (ordinary one-way ANOVA with Holm–Sidak’s multiple comparisons, *p* < 0.0001). Analysis of the re-learning dynamics revealed (Figure 6b) that the DAT-KO rats performed tasks significantly worse than the WT rats: to reach the learning criterion, they needed significantly more days (Log-rank Mantel–Cox test, *p* < 0.0001). The control group was fully re-trained in 7 days, whereas for knockout rats, re-training took 12 days.

**Figure 7:**
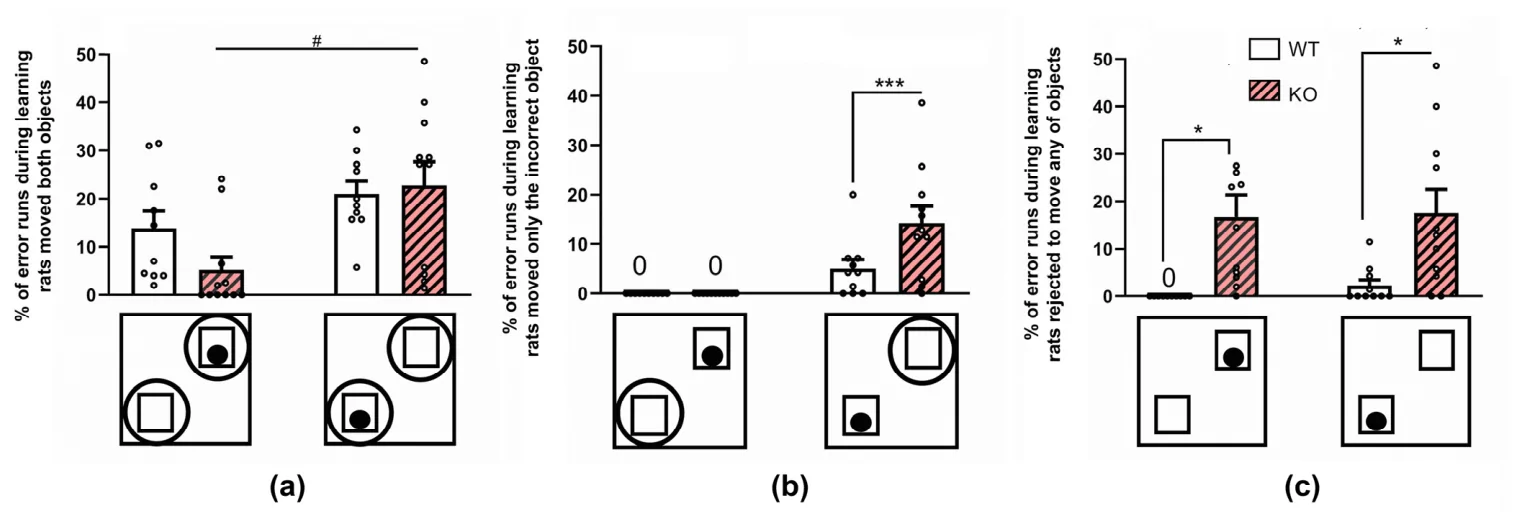
Comparison of the percentage of error runs during learning and re-learning in WT (n = 10) and DAT-KO (n = 11) rats when the spatial position of the objects was changed; (**a**) animals moved both objects; (**b**) animals moved only the incorrect object and (**c**) animals refused to move any of objects. Results are presented as the mean ± SEM. # *p* < 0.05; * *p* < 0.05; *** *p* < 0.001; Kruskal–Wallis test with Dunn’s multiple comparisons. The designation of reinforced and non-reinforced objects is as shown in Figure 2. The object selection option is marked by a circle.

An analysis of the errors made by rats during re-learning showed very important differences. By comparing the data obtained during learning and re-learning sessions, we found that the frequency of first-type errors (i.e., animals moved both objects) remained practically unchanged for WT rats (13.85% and 21%, respectively, Kruskal–Wallis test with Dunn’s multiple comparisons, *p* = 0.195), whereas it increased for DAT-KO rats (from 5.19% to 22.73%, *p* < 0.05, Kruskal–Wallis test with Dunn’s multiple comparisons, *p* = 0.004) (Figure 7a). Furthermore, in contrast to Experiments 1 and 2, in Experiment 3, i.e., the re-learning phase, the number of second-type errors when animals moved the non-reinforced distant object increased (Figure 7b). Such errors occurred significantly more often in DAT-KO rats (Kruskal–Wallis test with Dunn’s multiple comparisons, *p* < 0.0001).

It should be noted that errors of the third type, in which the rat did not move any of the presented objects and did not receive food reinforcement, were almost exclusively characteristic of DAT-KO rats (Figure 7c). There were significantly more errors in the DAT-KO rats than in the WT rats during both the learning stage (Kruskal–Wallis test with Dunn’s multiple comparisons, *p* = 0.010) and the re-learning (Kruskal–Wallis test with Dunn’s multiple comparisons, *p* = 0.016).

Analysis of the learning and re-learning periods showed that DAT-KO rats were able to learn the behavioral task at the same level as WT rats, regardless of the shape of the objects presented. However, they performed the task significantly worse than the control rats in re-learning and remembering the new location of the rewarded object.

## 4. Discussion

The aim of the present study was to reveal the effect of object shape and position on spatial learning and re-learning in DAT knockout rats, which are characterized by pronounced hyperdopaminergia due to the inability to reuptake dopamine [34].

It should be noted that in the present experiments, as in our previous works [27,33], DAT-KO rats were more hyperactive than control animals, which became evident when comparing the distances traveled and the time spent in the arena during task performance. Importantly, during learning and re-learning, these parameters were similar in control rats; in DAT-KO rats, they decreased during re-learning. These findings likely reflect the nature of hyperactive rats’ adaptation to the environment, because a decline in hyperactivity leads to faster food searching. The control rats performed tasks very quickly, and it was not possible for them to perform tasks within a shorter period of time.

Within the RedBox testing apparatus, animals were trained to recognize the shape and location of rewarded objects. During learning, DAT-KO and control WT rats reached the learning criterion independently of the object shape and location within the same period of time (Figure 3a,b). This fact is quite surprising, because it is well known that abnormal functioning of the dopaminergic system leads to disturbances in spatial memory in humans and animals [12,44]. In our previous studies [36,45], we described the inability of DAT-KO rats to perform spatial task at the same level as control animals. It was found that in spatial non-manipulative tasks in an 8-arm radial maze, DAT-KO rats demonstrated fewer correct runs than WT animals alongside impairment in spatial working memory [45]. Knockout rats used behavioral tactics different from those in control rats, and their tactics led to less successful task performance and, consequently, less obtainment of rewards [41]. In the Hebb–Williams maze, performance of behavioral spatial memory tasks in DAT-KO rats was significantly worse than in control WT rats [36,37,38]. When solving the spatial memory task, DAT-KO rats spent more time on task performance, made more entries to the error zones, and made significantly more erroneous returns to the start [41]. All these data suggest that solving spatial tasks is very complex under disturbances in DA regulation. This is not surprising, since patients with DA-related disorders also have difficulty performing spatial memory tasks [8,46]. Nevertheless, including objects into the behavioral paradigm in our experimental protocol led to a marked improvement in the ability of mutant rats to perform spatial tasks correctly at the same level as control animals. In previous experiments, we observed a positive effect of object manipulation on long-term memory when rats were required to recognize familiar and novel objects, receiving reinforcement only for moving familiar objects [40]. However, the spatial position of all objects was identical. In the present study, using manipulative skills for remembering rewarded objects’ spatial location was very fruitful for successful training.

One more important result of our experiments was that both DAT-KO and WT rats could easily transfer their ability to manipulate by a reinforced object cube, located on a special position to another geometric object, pawn (Figure 4a,b). It should be noted that the level of correct runs was approximately the same in both groups. Thus, it should be noted that the shape of the object did not affect the accuracy of task performance.

One possible explanation for these results may be the peculiarities of spatio-motor information acquisition by DAT-KO rats. The additional information to remember may slow down the rate of hyperactive rats’ runs and thus increase their ability to remember task rules. The absence of the influence of object shape on task performance may be connected with the stable acquisition of task rules, and only minor corrections for successful performance may be needed.

The marked differences in task performance between knockout and control animals were found at the re-learning stage. The most prominent results were obtained by comparing different types of errors during learning and re-learning. During re-learning, DAT-KO rats moved a non-reinforced object which had been rewarded previously more often than control rats. This fact reflects a high level of inflexibility in DAT-KO rats, which is connected to their marked perseverative reactions caused by hyperactivity [39]. These perseverative reactions are key characteristics of DAT-KO rats. It was found that conditioned associative learning was impaired in DAT knockout rats [47]. In addition, DAT-KO rats demonstrate cognitive impairments in spatial tasks involving re-learning in a T-maze [39].

The role of the DA in the existence of adaptive memory was proposed as crucial brain system for long-term memory [48]. It should be mentioned that cortico-striatal connections may be disrupted after long-term DA signaling alterations [49]. In numerous investigations, the DA system was found to have an important role in memory traces’ consolidation [50,51,52,53]. We propose that the abnormal structural relationships in the brain of DAT-KO rats form the basis of observed impairments. The prefrontal cortex DA was found to have an influence on striatal activity under instrumental learning and to differ in male and female animals [54]. Using an event-related fMRI method, it was found that interactions between the prefrontal cortex and basal ganglia may lead to an inhibition of the ongoing behavior required to complex tasks well and flexibly [55]. It was found that excessive DA in DAT-KO mice impairs the induction of long-term potentiation in the PFC, which may prevent the formation of long-term memory traces [56]. The prefrontal cortex is a leading structure in the organization of goal-directed behavior [57]. It is important for the realization of complex behavior and spatial orientation [58,59]. It is likely that the DA level in PFC in both control and DAT-KO rats is sufficient for correct task acquisition and solution during learning.

It should be noted that neurons projecting from the medial PFC to striatum are involved in spatial working memory [60]. Not long ago, the role of the medial PFC in mice’s spatial working memory was revealed. It was proposed that the medial PFC encodes spatial positions and the variables affecting them [59]. Thus, these connections provide spatial orientation and motor control regulation, but in hyperdopaminergic rats, these influences are not sufficient for fast improvement of their behavior when task rules change for re-learning.

Rats lacking DAT have morphological and functional abnormalities in the brain [61]. A significant loss of volume in the dorsal striatum was observed. Conversely, an increase in volume was observed in the prefrontal and midbrain regions. The authors attribute these abnormalities to adaptive changes in the rat’s brain resulting from DAT blockade starting in the prenatal period, which leads to motor hyperactivity and impairments in learning and memory. Disruptions of the connectivity between the striatum and PFC, which mediate spatial orientation and motor control, lead to behavioral errors in hyperdopaminergic DAT-KO rats when task rules are changed during re-learning. These data allow us to think that cortico-striatal connections play a pivotal role in learning in DAT knockout rats due to abnormal dopamine system function [61].

The results of our previous experiments [35,37,40] confirm the hypothesis that the spatial learning strategies employed by hyperactive DAT-KO rats differ from those used by control rats. In this study, we demonstrated that introducing additional objects into the experimental environment facilitates successful adaptation during spatial foraging tasks. This finding could potentially be used as the basis for the development of new methods, which differ from adopted approaches, of teaching children with ADHD. We hypothesize that combining manipulative activity with mental representations in training tasks could help children and adolescents with ADHD to improve their abilities.

The literature and data obtained during our experiments should be interpreted in light of certain limitations. Firstly, both the knockout and control groups of rats underwent similar phases of the behavioral experiment and acquired new skills during the training and re-training phases. However, we only compared the behavior parameters of all groups of rats during the same phases, when they had the same experience. To some extent, this approach limits the findings of our study. Moreover, only male knockout rats and wild-type counterparts were used in our experiments. Further experiments involving females may reveal new behavioral characteristics of hyperactive DAT-KO rats with hyperdopaminergia and would allow for a deeper understanding of the mechanisms underlying dopaminergic dysfunction in this animal model.

## 5. Conclusions

Our findings provide an opportunity to consider that introducing new objects into the task paradigm could improve the spatial memory of DAT-KO rats and help them to adapt to the conditions of the task. It seems essential to combine new spatial object arrangements with the need to manipulate them for successful training. Any additional objects can be considered an enrichment of the experimental environment. Including objects in the task paradigm therefore represents an enrichment of the animal’s housing cage. Therefore, it is likely that the spatial memory of DAT-KO rats can be improved through such enrichment. The presence of common symptomatology between in mice and rats with dopamine transporter deficiency [33,34,62] and humans with ADHD [9,15,26] suggests that these animals may serve as a useful animal model for testing new treatments for ADHD and possibly other hyperkinetic disorders. We hypothesize that incorporating manipulative skills into spatial learning could be an effective method of creating behavioral tasks for patients with dopamine transmission disorders. However, new tasks and forms of enrichment are required to confirm this hypothesis.

## Data Availability

The original contributions presented in this study are included in the article material. Further inquiries can be directed to the corresponding author.

## Abbreviations

The following abbreviations are used in this manuscript:

DA	Dopamine
DAT	Dopamine transporter
DAT-KO	Dopamine transporter knockout animals
WT	Wild-type animals
ADHD	Attention deficit hyperactivity disorder
PFC	Prefrontal cortex
